# Segmentation of High Dimensional Time-Series Data Using Mixture of Sparse Principal Component Regression Model with Information Complexity

**DOI:** 10.3390/e22101170

**Published:** 2020-10-17

**Authors:** Yaojin Sun, Hamparsum Bozdogan

**Affiliations:** Department of Business Analytics and Statistics, University of Tennessee, Knoxville, TN 37996, USA; ysun52@vols.utk.edu

**Keywords:** high dimensional time-series, segmentation, mixture regression, sparse PCA, entropy-based robust EM, information complexity criteria

## Abstract

This paper presents a new and novel hybrid modeling method for the segmentation of high dimensional time-series data using the mixture of the sparse principal components regression (*MIX-SPCR*) model with information complexity (ICOMP) criterion as the fitness function. Our approach encompasses dimension reduction in high dimensional time-series data and, at the same time, determines the number of component clusters (i.e., number of segments across time-series data) and selects the best subset of predictors. A large-scale Monte Carlo simulation is performed to show the capability of the *MIX-SPCR* model to identify the correct structure of the time-series data successfully. *MIX-SPCR* model is also applied to a high dimensional Standard & Poor’s 500 (S&P 500) index data to uncover the time-series’s hidden structure and identify the structure change points. The approach presented in this paper determines both the relationships among the predictor variables and how various predictor variables contribute to the explanatory power of the response variable through the sparsity settings cluster wise.

## 1. Introduction

This paper presents a new and novel method for the segmentation and dimension reduction in high dimensional time-series data. We develop hybrid modeling between *mixture-model cluster analysis* and *sparse principal components regression* (MIX-SPCR) model as an expert unsupervised classification methodology with *information complexity* (ICOMP) criterion as the fitness function. This new approach performs dimension reduction in high dimensional time-series data and, at the same time, determines the number of component clusters.

The research of time-series segmentation and change point positioning has been a hot topic of research for a long time. Different research groups have provided solutions with various approaches in this area, including, but not limited to, Bayesian methods Barber et al. [1], fuzzy systems Abonyi and Feil [2], and complex system modeling Spagnolo and Valenti [3], Valenti et al. [4], S Lima [5], Ding et al. [6]. We group these approaches into two branches, one based on complex systems modeling and the other on the statistical model through parameter estimation and inference. Among the complex systems-based modeling approaches, it is worth noting a series of papers that use the stochastic volatility model by Spagnolo and Valenti [3]. For example, these authors used a nonlinear Hestone model to analyze 1071 stocks on the New York Stock Exchange (1987–1998). After accounting for the stochastic nature of volatility, the model is well suited to extracting the escape time distribution from financial time-series data. The authors also identified the NES (Noise Enhanced Stability) effect to measure market dynamics’ stabilizing effect. The approach we propose in this paper belongs to another branch of using a statistical model on time scales. Along with the empirical analysis, we show a broader view of how different companies/sectors behaved across different periods. In particular, we use a mixture-model based statistical methodology to segment the time-series and determine change points.

The mixture-model cluster analysis of regression models is not new. These models are also known as *“cluster-wise regression”*, *“latent models”*, and *“latent structure models of choice”*. These models have been well-studied among statisticians, machine learning researchers, and econometricians in the last several decades to construct time-series segmentation models and identify change points. They have many useful theoretical and applied properties. Mixture-model cluster analysis of regression models is a natural extension of the standard multivariate Gaussian mixture-model cluster analysis. These models are beneficial to study heterogeneous data sets that involve not just one response variable but can have several responses or target-dependent variables simultaneously with a given set of independent variables. Recently, they have been proven to be a precious class of models in various disciplines in *behavioral and economic research*, *ecology*, *financial engineering*, *process control*, *and monitoring*, *market research*, *transportation systems*. Additionally, we also witness the mixture model’s usage in the *analysis of scanner panel*, *survey, and other choice data to study consumer choice behavior and dynamics*  Dillon et al. [7].

In reviewing the literature, we note that Quandt and Ramsey [8] and Kiefer [9] studied data sets by applying a mixture of two regression models using moment generating function techniques to estimate the unknown model parameters. Later, De Veaux [10] developed an EM algorithm to fit a mixture of two regression models. DeSarbo and Cron [11] used similar estimating equations and extended the earlier work done on a mixture of two regression models to a mixture of K-component regression models. For an excellent review article on this problem, we refer the reviewers to Wedel and DeSarbo [12].

In terms of these models’ applications in the segmentation of time-series, they can be seen in the early work of Sclove [13], where the author applied the mixture model to the segmentation of US gross national product, a high dimensional time-series data. Specifically, Sclove [13] used the statistical model selection criteria to choose the number of classes.

With the currently existing challenges in mind in the segmentation of time-series data, in this paper, our objective and goal are to develop a new methodology which can:

- Identify and select variables that are sparse in the *MIX-SPCR* model.
- Treat each time segment continuously in the process with some specified probability density function (pdf).
- Determine the number of time-series segments and the number of sparse variables and estimate the structural change points simultaneously.
- Develop a robust and efficient algorithm for estimating model parameters.

We aim to achieve these objectives by developing the information complexity (ICOMP) criteria as our fitness function throughout the paper for the segmentation of high-dimensional time-series data.

Our approach involves a two-stage procedure. We first make a variable selection by using SPCA with the benefit of sparsity. We then fit the sparse principal component regression (SPCR) model by transforming the original high dimensional data into several main principal components and estimating relationships between the sparse component loadings and the response variable. In this way, the mixture model not only handles the curse of dimensionality but also maintains the model’s excessive explanatory power. In this manner, we choose the best subset of predictors and determine the number of time-series segments in the *MIX-SPCR* model simultaneously using ICOMP.

The rest of the paper is organized as follows. In Section 2, we present the model and methods. In particular, we first briefly explain sparse principal component analysis (SPCA) due to Zou et al. [14] in Section 2.1. In Section 2.2, we modify SPCA and develop mixtures of the sparse principal component regression (*MIX-SPCR*) model for the segmentation of time-series data. In Section 3, we present a regularized entropy-based Expectation and Maximization (EM) clustering algorithm. As is well known, the EM algorithm performs through maximizing the likelihood of the mixture models. However, to make the conventional EM algorithm robust (not sensitive to initial values) and converge to global optimum, we use the robust version of the EM algorithm for the *MIX-SPCR* model based on the work of Yang et al. [15]. These authors addressed the robustness issue by adding an entropy term of mixture proportions to the conventional EM algorithm’s objective function. While our EM algorithm is in the same spirit of the Yang et al. [15] approach, there are significant differences between our approach and theirs. Yang’s robust EM algorithm merely deals with the usual clustering problem without involving any response (or dependent) variable or time factor in the data. We extend it to the case of the *MIX-SPCR* model in the context of time-series data. In Section 4, we discuss various information criteria, specifically the information complexity based criteria (ICOMP). We derive the ICOMP for the *MIX-SPCR* model based on Bozdogan’s previous research ([16,17,18,19,20]). In Section 5, we present our Monte Carlo simulation study. Section 5.2 involves an experiment on the detection of structural points, and Section 5.3 presents a large scale Monte Carlo simulation verifying the advantage of the *MIX-SPCR* with statistical information criteria. We provide a real data analysis in Section 6 using the daily adjusted closing S&P 500 index and stock prices from the Yahoo Finance database that spans the period from January 1999 to December 2019. Finally, our conclusion and discussion are presented in Section 7.

## 2. Model and Methods

In this section, we briefly present the *sparse principal component analysis* (*SPCA*), *sparse principal component regression* (*SPCR*) as a background. Then, by hybridizing these two methods within the mixture model, we propose the *mixture-model cluster analysis of sparse principal component regression* (abbreviated as *MIX-SPCR* model hereafter), for segmentation of high dimensional time-series datasets. Compared with a simple linear combination of all explanatory variables (i.e., the dense PCA model), the new approach interprets better because it maintains a sparsity specification.

**Figure 1:**
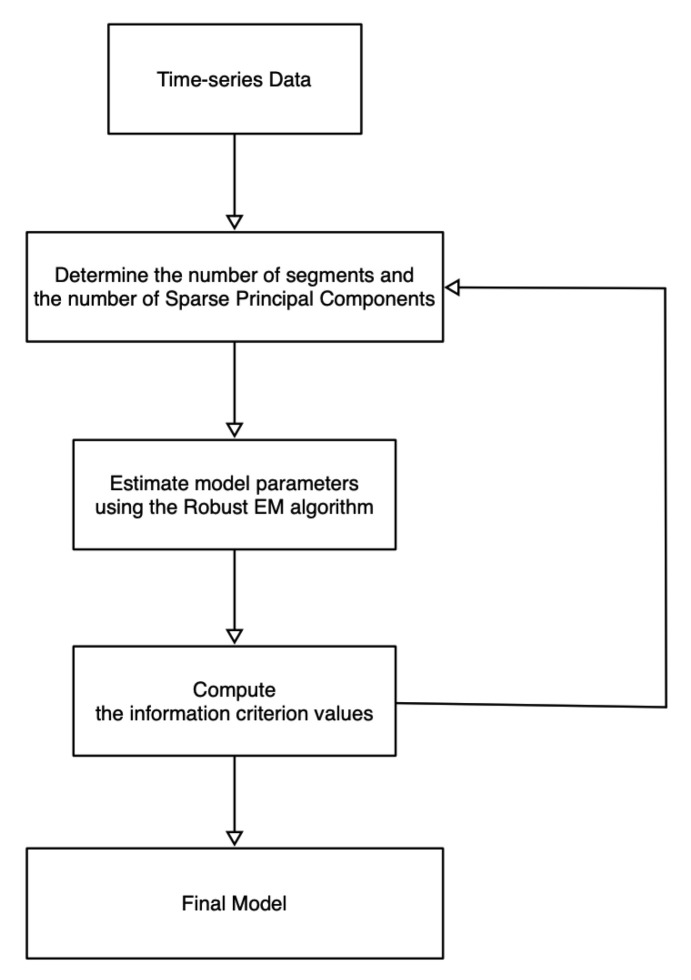
The flowchart of the MIX-SPCR method.

Referring to Figure 1, we first show the overall structure of the model in this paper. The overall processing flow is that we clean and standardize the data after obtaining the time-series data. Subsequently, we specify the number of time-series segments and how many Sparse Principal Components (SPCs) each segment contains. Using the Robust EM algorithm (Section 3), we estimate the model parameters, especially the boundaries (also known as *change points*) of each time segment. The information criterion values are calculated using the method of Section 4. By testing different numbers of time segments/SPCs, we obtain multiple criterion values. According to the calculated information criterion values, we choose the most appropriate model with the estimated parameters.

### 2.1. Sparse Principal Component Analysis (SPCA)

Given the input data matrix, X with *n* number of observations and *p* variables, we decompose X using the singular value decomposition (SVD). We write the decomposition procedure as $X=UDV^{T}$, where D is a diagonal matrix of singular values and orthogonal columns U and V as the left and right singular vectors. When we perform SVD of a data matrix X that has been centered, by subtracting each column’s mean, the process is the well-known *principal component analysis* (*PCA*). As discussed by Zou et al. [14], PCA has several advantages as compared with other dimensionality reduction techniques. For example, the PCA can sequentially identify the source of variability by considering the linear combination of all the variables. Because of the orthonormal constraint during the computation, all the calculated *principal components* (*PCs*) have clear geometrical interpretation corresponding to the original data space as a dimension reduction technique. Because PCA can deal with *“the curse of dimensionality”* of high-dimensional data sets, it has been widely used in real-world scenarios, including biomedical and financial applications.

Even though PCA has excellent properties that are desirable in real-world applications and statistical analysis, the interpretation of PCs is often difficult since it includes all the variables as linear combinations of all the original variables in each of the PCs. In practice, the principal components always have a large number of non-zero coefficient values for corresponding variables. To resolve this drawback, researchers proposed various improvements focusing on PCA’s sparsity while maintaining the minimal loss of information. Shen and Huang [21] designed an algorithm to iteratively extract top PCs using the so-called *penalized least sum of square* (*PLSS*) criterion. Zou et al. [14] utilized the lasso penalty (via Elastic Net) to maintain a sparse loading of the principal components, which is named *sparse principal component analysis* (*SPCA*).

In this paper, we use the sparse principal component analysis (SPCA) proposed by Zou et al. [14]. Given the data matrix X, we minimize the objective function to obtain the SPCA results:

(1) $$\begin{array}{c} \left(\hat{A},\hat{B}\right)=\underset{A,B}{\arg\min}\sum_{i=1}^{n}{∥x_{i}^{T}-{\mathrm{AB}}^{T}x_{i}^{T}∥}^{2}+\sum_{j=1}^{k}\lambda_{1,j}{∥B_{\left(j\right)}∥}_{1}+\lambda_{2}\sum_{j=1}^{k}{∥B_{\left(j\right)}∥}_{2}^{2}, \end{array}$$

subject to

(2) $$\begin{array}{c} A^{T}A=I_{k}. \end{array}$$

where $I_{k}$ is the identity matrix. We maintain the hyperparameters $\lambda_{1,j}$ and $\lambda_{2}$ to be non-negative. The A and B matrices of size (p×k) are given by

(3) $$\begin{array}{c} B=\left[\begin{array}{ccc} B_{1,1} & \cdots & B_{1,k}\\ \vdots & \ddots & \vdots \\ B_{p,1} & \cdots & B_{p,k} \end{array}\right]=\left[B_{\left(1\right)}|\ldots |B_{\left(k\right)}\right]=\left[\begin{array}{c} B_{1}\\ \vdots \\ B_{p} \end{array}\right], \end{array}$$

and

(4) $$\begin{array}{c} A=\left[\begin{array}{ccc} A_{1,1} & \cdots & A_{1,k}\\ \vdots & \ddots & \vdots \\ A_{p,1} & \cdots & A_{p,k} \end{array}\right]=\left[A_{\left(1\right)}|\ldots |A_{\left(k\right)}\right]=\left[\begin{array}{c} A_{1}\\ \vdots \\ A_{p} \end{array}\right]. \end{array}$$

If we choose the first *k* principal components from the data matrix X, then the estimate ${\hat{B}}_{\left(j\right)}$ contains the sparse loading vectors, which are no longer orthogonal.

A bigger $\lambda_{1,j}$ means a greater penalty for having non-zero entries in ${\hat{B}}_{\left(j\right)}$. By using different $\lambda_{1,j}$, we control the number of zeros in the *j*th loading vector. If $\lambda_{1,j}=0$ for j=1,2,…,k, this problem reduces to usual PCA.

Zou et al. [14] proposed a generalized SPCA algorithm to solve the optimization problem in Equation (1). The algorithm applies the Elastic Net (EN) to estimate $B_{\left(j\right)}$ iteratively and update matrix A. However, this algorithm is not the only available approach for extracting principal components with sparse loadings. The SPCA could also be computed through dictionary learning by Mairal et al. [22]. By introducing the probability model of principal component analysis, SPCA is equivalent to the *sparse probabilistic principal component analysis* (*SPPCA*) if the prior is Laplacian distribution for each weight matrix element (Guan and Dy [23], Williams [24]). For further discussion on SPPCA, we refer readers to those related publications for more details.

Next, we introduce the *MIX-SPCR* model for the segmentation of time-series data.

### 2.2. Mixtures of SPCR Model for Time-Series Data

Suppose the continuous response variable is denoted as $y=\left\{y_{i}|1\leq i\leq n\right\}$, where *n* represents the number of observations (time points). Similarly, we have the predictors denoted as $X=\left\{x_{i}|1\leq i\leq n\right\}$. Each observation $x_{i}$ has *p* dimensions and is represented as $x_{i}={\left[x_{1,i},x_{2,i},\cdots ,x_{p,i}\right]}^{T}$. Both the response variable and independent variables are collected sequentially labeled by time points $T=\left[t_{1},t_{2},\cdots ,t_{n}\right]$.

The finite mixture model allows applying cluster analysis on conditionally dependent data into several classes. In the time-series data scenario, researchers cluster the data $\left(\left(t_{1},x_{1},y_{1}\right),\left(t_{2},x_{2},y_{2}\right),\cdots ,\left(t_{n},x_{n},y_{n}\right)\right)$ into several homogeneous groups where the number of groups *G* is unknown in general. Within each group, we apply the SPCA to extract top *k* principal components that each of them has a sparse loading of *p* variable coefficients. The extracted top *k* PCs are denoted as matrix $P_{p\times k}$. We also use $P_{g}$ to represent the principal component matrix obtained from the group indexed by g=1,2,…,G.

The SPCR model assumes that each pair $\left(x_{i},y_{i}\right)$ is independently drawn from a cluster using both the SPCA and the regression model as follows.

(5) $$\begin{array}{c} y_{i}=x_{i}^{T}P_{g}\beta_{g}+\epsilon_{i,g},i=1,2,\cdots ,n, \end{array}$$

where $\beta_{g}={\left[\beta_{g,1},\beta_{g,2},\cdots ,\beta_{g,k}\right]}^{T}$.

For each group *g*, the random error is assumed to be Gaussian distributed. That is, $\epsilon_{i,g}∼N\left(0,\sigma_{g}^{2}\right)$. If the response variable is multivariate, then the random error is usually also assumed to be a multivariate Gaussian distribution. Thus the probability density function (pdf) of the SPCR model is

(6) $$\begin{array}{c} f\left(y_{i}|x_{i},P_{g},\beta_{g}\right)=N\left(y_{i}|x_{i}^{T}P_{g}\beta_{g},\sigma_{g}^{2}\right). \end{array}$$

We emphasize here that the noise (i.e., the error term) included in the statistical model is drawn from a normal distribution independent for each time-series segment, with different values of $\sigma_{g}^{2}$ for each period. Since we use the EM algorithm to estimate the parameters of the model, the noise parameter $\sigma_{g}^{2}$ can be estimated accurately as well. Future studies will consider introducing different noise distributions, such as α-stable Lévy noise [25], and other non-Gaussian noise distributions to further extend the current model.

We also consider time factor $t_{i}$ in the SPCR model of time-series data to be continuous. The pdf of the time factor is

(7) $$\begin{array}{c} f\left(t_{i}|v_{g},\sigma_{g}^{2,\mathrm{time}}\right)=N\left(t_{i}|v_{g},\sigma_{g}^{2,\mathrm{time}}\right), \end{array}$$

where $v_{g}$ is the mean, and $\sigma_{g}^{2,\mathrm{time}}$ is the variance of the time segment *g*. Apart from the normal distribution, our approach can also be generalized to other distributions for the time factor, such as skewed distributions, Student’s t-distribution, ARCH, GARCH time-series models, and so on.

As a result, if we use the *MIX-SPCR* model to perform segmentation of time-series data, the likelihood function of the whole data $\left(\left(t_{1},x_{1},y_{1}\right),\left(t_{2},x_{2},y_{2}\right),\cdots ,\left(t_{n},x_{n},y_{n}\right)\right)$ with *G* number of clusters (or segments) is given by

(8) $$\begin{array}{c} L=\prod_{i=1}^{n}\prod_{g=1}^{G}{\left[\pi_{g}f\left(y_{i}|x_{i},P_{g},\beta_{g}\right)f\left(t_{i}|v_{g},\sigma_{g}^{2,\mathrm{time}}\right)\right]}^{z_{g,i}}, \end{array}$$

where the $\pi_{g}$ is the mixing proportion with the constraint that $\pi_{g}\geq 0$ and $\sum_{g=1}^{G}\pi_{g}=1$. We follow the definition of missing values by Yang et al. [15] and let $Z=\left\{Z_{1},Z_{2},\cdots ,Z_{n}\right\}$. If $Z_{i}=g$, then $z_{g,i}=1$, otherwise, $z_{g,i}=0$. Then the log-likelihood function of the *MIX-SPCR* model models is

(9) $$\begin{array}{cc} L_{\mathrm{mix}} & =\log\left(L\right)\\ \; & =\sum_{i=1}^{n}\sum_{g=1}^{G}z_{g,i}\log\left[\pi_{g}f\left(y_{i}|x_{i},P_{g},\beta_{g}\right)f\left(t_{i}|v_{g},\sigma_{g}^{2,\mathrm{time}}\right)\right]\\ \; & =\sum_{i=1}^{n}\sum_{g=1}^{G}z_{g,i}\left[\log\;\pi_{g}+\log\;f\left(y_{i}|x_{i},P_{g},\beta_{g}\right)+\log\;f\left(t_{i}|v_{g},\sigma_{g}^{2,\mathrm{time}}\right)\right] \end{array}$$

(10) $$\begin{array}{cc} \; & =\underset{L_{\pi }}{\underset{︸}{\sum_{i=1}^{n}\sum_{g=1}^{G}z_{g,i}\log\;\pi_{g}}}+\underset{L_{\mathrm{SPCR}}}{\underset{︸}{\sum_{i=1}^{n}\sum_{g=1}^{G}z_{g,i}\log\;f\left(y_{i}|x_{i},P_{g},\beta_{g}\right)}}+\underset{L_{\mathrm{time}}}{\underset{︸}{\sum_{i=1}^{n}\sum_{g=1}^{G}z_{g,i}\log\;f\left(t_{i}|v_{g},\sigma_{g}^{2,\mathrm{time}}\right)}}. \end{array}$$

We denote $z=\left[z_{g,i}\right]$ where g=1,2,⋯,G and i=1,2,⋯,n.

Given the number of segments, researchers usually apply the EM algorithm to determine the optimal segmentation by setting the objective function as $J_{\mathrm{EM}}=L_{\mathrm{mix}}$ (Gaffney and Smyth [26], Esling and Agon [27], Gaffney [28]).

## 3. Regularized Entropy-Based EM Clustering Algorithm

The EM algorithm is a method for iteratively optimizing the objective function. As discussed in Section 2.2, by setting the objective function as the log-likelihood function, we can use the EM algorithm to identify optimal segmentation of time series.

However, in practice, the EM algorithm is sensitive to model initialization conditions and cannot estimate the number of clusters appropriately. To deal with the initialization problem, in 2012, Yang et al. [15] proposed using an entropy penalty to stabilize the computation of each step. The improved method is called the *robust EM algorithm*. In this paper, we extend the robust EM algorithm to deal with time-series data for the *MIX-SPCR*  model.

In Section 3.1, we discuss the entropy term of the robust EM algorithm. Then, we show the extension of the robust EM algorithm for the *MIX-SPCR* model in Section 3.2 and Section 3.3.

### 3.1. The Entropy of EM Mixture Probability

As introduced in Equation (8), the $\pi_{g}$ represents the mixture probability of each cluster or segment. In other words, the value of $\pi_{g}$ is the probability that a data point belongs to group *g*. The clustering complexity is determined by the number of clusters and corresponding probability values, which could be obtained using entropy. Given $\left\{\pi_{g}|1\leq g\leq G\right\}$, the entropy of $Z_{i}$ is

(11) $$\begin{array}{c} H\left(Z_{i}|\left\{\pi_{g}|1\leq g\leq G\right\}\right)=-\sum_{g=1}^{G}\pi_{g}log\left(\pi_{g}\right),\mathrm{for}\;i=1,2,\cdots ,n. \end{array}$$

Then the entropy of Z is written as,

(12) $$\begin{array}{cc} H(Z|\left\{\pi_{g}|1\leq g\leq G\right\}) & =\sum_{i=1}^{n}H\left(Z_{i}|\left\{\pi_{g}|1\leq g\leq G\right\}\right)\\ \; & =-\sum_{i=1}^{n}\sum_{g=1}^{G}\pi_{g}log\left(\pi_{g}\right)\\ \; & =-n\sum_{g=1}^{G}\pi_{g}log\left(\pi_{g}\right). \end{array}$$

The objective function of the robust EM algorithm is

(13) $$\begin{array}{c} J_{\mathrm{Robust}-\mathrm{EM}}=L_{\mathrm{mix}}-\lambda_{\mathrm{Robust}-\mathrm{EM}}H\left(Z|\left\{\pi_{g}|1\leq g\leq G\right\}\right), \end{array}$$

where $\lambda_{\mathrm{Robust}-\mathrm{EM}}\geq 0$. The log-likelihood term $L_{\mathrm{mix}}$ is from Equation (9), which gives the goodness-of-fit.

Next, we present the steps of the EM algorithm for maximizing the objective function in Equation (13).

### 3.2. E-Step (Expectation)

From a Bayesian perspective, we let ${\hat{z}}_{g,i}$ denote the posterior probability of the true cluster membership that a dataset triplet $\left(t_{i},x_{i},y_{i}\right)$ is drawn from group *g*. Using the Bayes theorem, we have

(14) $$\begin{array}{cc} {\hat{z}}_{g,i} & =E(Z_{i}=g|y_{i},x_{i},P_{g},\beta_{g}) \end{array}$$

(15) $$\begin{array}{cc} \; & =\frac{\pi_{g}N\left(y_{i};x_{i}P_{g}\beta_{g},\sigma_{g}^{2}\right)N\left(t_{i}|v_{g},\sigma_{g}^{2,\mathrm{time}}\right)}{\sum_{h=1}^{G}\pi_{h}N\left(y_{i};x_{i}P_{h}\beta_{h},\sigma_{h}^{2}\right)N\left(t_{i}|v_{h},\sigma_{h}^{2,\mathrm{time}}\right)}. \end{array}$$

### 3.3. M-Step (Maximization)

Using the robustified derivation of ${\hat{\pi }}_{g}$, the estimated mixture proportion, we have

(16) $$\begin{array}{c} {\hat{\pi }}_{g}^{\mathrm{new}}={\hat{\pi }}_{g}^{\mathrm{EM}}+{\hat{\lambda }}_{\mathrm{Robust}-\mathrm{EM}}{\hat{\pi }}_{g}^{\mathrm{old}}\left(log\left({\hat{\pi }}_{g}^{\mathrm{old}}\right)-\sum_{h=1}^{G}\left({\hat{\pi }}_{h}^{\mathrm{old}}\log\left({\hat{\pi }}_{h}^{\mathrm{old}}\right)\right)\right), \end{array}$$

where

(17) $$\begin{array}{c} {\hat{\pi }}_{g}^{\mathrm{EM}}=\frac{\sum_{i=1}^{n}{\hat{z}}_{g,i}}{n}. \end{array}$$

We follow the recommendation of Yang et al. [15] for the value of ${\hat{\lambda }}_{\mathrm{Robust}-\mathrm{EM}}^{\mathrm{new}}$ as

(18) $$\begin{array}{c} {\hat{\lambda }}_{\mathrm{Robust}-\mathrm{EM}}^{\mathrm{new}}=min\left\{\frac{\sum_{h=1}^{G}exp\left(-\eta n\left|{\hat{\pi }}_{g}^{\mathrm{new}}-{\hat{\pi }}_{g}^{\mathrm{old}}\right|\right)}{G},\frac{1-max\left\{\sum_{i=1}^{n}{\hat{z}}_{h,i}^{\mathrm{old}}/n|h=1,2,\cdots ,G\right\}}{-max\left\{{\hat{\pi }}_{h}^{\mathrm{old}}|h=1,2,\cdots ,G\right\}\sum_{h=1}^{G}{\hat{\pi }}_{h}^{\mathrm{old}}\log{\hat{\pi }}_{h}^{\mathrm{old}}}\right\}, \end{array}$$

where

(19) $$\begin{array}{c} \eta =min\left\{1,0.5^{\left\lfloor p/2-1\right\rfloor }\right\}, \end{array}$$

and *p* is the number of variables in the model.

We iterate E-step and M-step several times until convergence to obtain the parameter estimates. In particular, the $\beta_{g}$ values get updated by maximizing the $J_{\mathrm{Robust}-\mathrm{EM}}$ from Equation (13). Since we fix the number of segments and principal components during each E-step and M-step, the updated values of $\beta_{g}$ and $\sigma_{g}$ can be calculated using $L_{\mathrm{mix}}$ directly. The estimated values of $\beta_{g}$ and $\sigma_{g}$ are given as follows.

$$\begin{array}{cc} {\hat{\beta }}_{g}^{\mathrm{new}} & ={\left[\sum_{i=1}^{n}{\hat{z}}_{g,i}^{\mathrm{old}}{\left(x_{i}^{T}P_{g}\right)}^{T}\left(x_{i}^{T}P_{g}\right)\right]}^{-1}\sum_{i=1}^{n}{\hat{z}}_{g,i}^{\mathrm{old}}{\left(x_{i}^{T}P_{g}\right)}^{T}y_{i} \end{array}$$

(20) $$\begin{array}{cc} \; & ={\left[\sum_{i=1}^{n}{\hat{z}}_{g,i}^{\mathrm{old}}P_{g}^{T}x_{i}x_{i}^{T}P_{g}\right]}^{-1}\sum_{i=1}^{n}{\hat{z}}_{g,i}^{\mathrm{old}}P_{g}^{T}x_{i}y_{i}, \end{array}$$

(21) $$\begin{array}{cc} {\hat{\sigma }}_{g}^{2,\mathrm{new}} & =\sum_{i=1}^{n}{\hat{z}}_{g,i}^{\mathrm{old}}{∥y_{i}-x_{i}^{T}P_{g}{\hat{\beta }}_{g}^{\mathrm{new}}∥}_{2}^{2}/\sum_{i=1}^{n}{\hat{z}}_{g,i}^{\mathrm{old}}. \end{array}$$

For the time factor, the estimated mean ${\hat{v}}_{g}$ and variance ${\hat{\sigma }}_{g}^{2,\mathrm{time}}$ are

(22) $$\begin{array}{cc} {\hat{v}}_{g} & =\frac{\sum_{i=1}^{n}{\hat{z}}_{g,i}t_{i}}{\sum_{i=1}^{n}{\hat{z}}_{g,i}}, \end{array}$$

(23) $$\begin{array}{cc} {\hat{\sigma }}_{g}^{2,\mathrm{time}} & =\frac{\sum_{i=1}^{n}{\hat{z}}_{g,i}{\left(t_{i}-{\hat{v}}_{g}\right)}^{2}}{\sum_{i=1}^{n}{\hat{z}}_{g,i}}. \end{array}$$

As discussed above, our approach is flexible in considering other distributional models for the time-series factor, which we will pursue in separate research work.

## 4. Information Complexity Criteria

Recently, the statistical literature recognized the necessity of introducing model selection as one of the technical areas. In this area, the entropy and the Kullback–Leibler [29] information (or KL distance) play a crucial role and serve as an analytical basis to obtain the forms of model selection criteria. In this paper, we use information criteria to evaluate a portfolio of competing models and select the best-fitting model with minimum criterion values.

One of the first information criteria for model selection in the literature is due to the seminal work of Akaike [30]. Following the entropy maximization principle (EMP), Akaike developed the Akaike’s Information Criterion (AIC) to estimate the expected KL distance or divergence. The form of AIC is

(24) $$\begin{array}{c} \mathrm{AIC}=-2\log L(\hat{\theta })+2k, \end{array}$$

where $L(\hat{\theta })$ is the maximized likelihood function, and *k* is the number of estimated free parameters in the model. The model with minimum AIC value is chosen as the best model to fit the data.

Motivated by Akaike’s work, Bozdogan [16,17,18,19,20,31] developed a new information complexity (ICOMP) criteria based on Van Emden’s [32] entropic complexity index in parametric estimation. Instead of penalizing the number of free parameters directly, ICOMP penalizes the covariance complexity of the model. There are several forms of ICOMP. In this section, we present the two general forms of ICOMP criteria based on the estimated inverse Fisher information matrix (IFIM). The first form is

(25) $$\begin{array}{cc} \mathrm{ICOMP}(\mathrm{IFIM}) & =-2\log L\left(\hat{\theta }\right)+2C\left({\hat{\Sigma }}_{model}\right)\\ \; & =-2\log L\left(\hat{\theta }\right)+2C_{1}\left({\hat{F}}^{-1}\right), \end{array}$$

where $L(\hat{\theta })$ is the maximized likelihood function, and $C_{1}\left({\hat{F}}^{-1}\right)$ represents the entropic complexity of IFIM. We define $C_{1}\left({\hat{F}}^{-1}\right)$ as

(26) $$\begin{array}{c} C_{1}\left({\hat{F}}^{-1}\right)=\frac{s}{2}\log\left(\frac{tr{\hat{F}}^{-1}}{s}\right)-\frac{1}{2}\log\left|{\hat{F}}^{-1}\right|, \end{array}$$

and where $s=\mathrm{rank}({\hat{F}}^{-1})$. We can also give the form of $C_{1}\left({\hat{F}}^{-1}\right)$ in terms of eigenvalues,

(27) $$\begin{array}{c} C_{1}\left({\hat{F}}^{-1}\right)=\frac{s}{2}\log\left(\frac{{\bar{\lambda }}_{a}}{{\bar{\lambda }}_{g}}\right), \end{array}$$

where ${\bar{\lambda }}_{a}$ is the arithmetic mean of the eigenvalues, $\lambda_{1},\lambda_{2},\ldots ,\lambda_{s}$, and ${\bar{\lambda }}_{g}$ is the geometric mean of the eigenvalues.

We note that ICOMP penalizes the lack of parsimony and the profusion of the model’s complexity through IFIM. It offers a new perspective beyond counting and penalizing number of estimated parameters in the model. Instead, ICOMP takes into account interaction (i.e., correlation) among the estimated parameters through the model fitting process.

We define the second form of ICOMP as

(28) $$\begin{array}{c} \mathrm{ICOMP}{\left(\mathrm{IFIM}\right)}_{C_{1F}}=-2\log L\left(\hat{\theta }\right)+2C_{1F}\left({\hat{F}}^{-1}\right), \end{array}$$

where $C_{1F}\left({\hat{F}}^{-1}\right)$ is given by

(29) $$\begin{array}{c} C_{1F}\left({\hat{F}}^{-1}\right)=\frac{s}{4}\frac{\frac{1}{s}tr\left({\left({\hat{F}}^{-1}\right)}^{T}\left({\hat{F}}^{-1}\right)\right)-{\left(\frac{tr\left({\hat{F}}^{-1}\right)}{s}\right)}^{2}}{{\left(\frac{tr\left({\hat{F}}^{-1}\right)}{s}\right)}^{2}}. \end{array}$$

In terms of the eigenvalues of IFIM, we write $C_{1F}\left({\hat{F}}^{-1}\right)$ as

(30) $$\begin{array}{c} C_{1F}\left({\hat{F}}^{-1}\right)=\frac{1}{4{\bar{\lambda }}_{a}^{2}}\sum_{j=1}^{s}{\left(\lambda_{j}-{\bar{\lambda }}_{a}\right)}^{2}. \end{array}$$

We want to highlight some features of $C_{1F}\left({\hat{F}}^{-1}\right)$ here. The term $C_{1F}\left({\hat{F}}^{-1}\right)$ is a second-order equivalent measure of complexity to the original term $C_{1}\left({\hat{F}}^{-1}\right)$. Additionally, we note that $C_{1F}\left({\hat{F}}^{-1}\right)$ is scale-invariant and $C_{1F}\left({\hat{F}}^{-1}\right)\geq 0$ with $C_{1F}\left({\hat{F}}^{-1}\right)=0$ only when all $\lambda_{j}={\bar{\lambda }}_{a}$. Furthermore, $C_{1F}\left({\hat{F}}^{-1}\right)$ measures the relative variation in the eigenvalues.

These two forms of ICOMP provide us an easy to use computational means in high dimensional modeling. Next, we derive the analytical forms of ICOMP in the *MIX-SPCR*  model.

### Derivation of Information Complexity in MIX-SPCR Model for Time-Series Data

We first consider the log-likelihood function of the *MIX-SPCR* model given in Equation (9),

(31) $$\begin{array}{c} L_{\mathrm{mix}}=L_{\pi }+L_{\mathrm{SPCR}}+L_{\mathrm{time}}. \end{array}$$

After some work, the estimated inverse Fisher information matrix (IFIM) of the mixture probabilities is

(32) $$\begin{array}{c} {\hat{F}}_{\pi }^{-1}=\left[\begin{array}{cccc} {\hat{\pi }}_{1}^{-1} & 0 & 0 & 0\\ 0 & {\hat{\pi }}_{2}^{-1} & 0 & 0\\ 0 & 0 & \ddots & 0\\ 0 & 0 & 0 & {\hat{\pi }}_{G}^{-1} \end{array}\right]. \end{array}$$

Similarly, for each segment *g*, the estimated IFIM, ${\hat{F}}_{g,\mathrm{SPCR}}^{-1}$, is

(33) $$\begin{array}{c} {\hat{F}}_{g,\mathrm{SPCR}}^{-1}=\left[\begin{array}{cc} {\hat{\sigma }}_{g}^{2}{\left[\sum_{i=1}^{n}{\hat{z}}_{g,i}{\left(x_{i}^{T}P_{g}\right)}^{T}\left(x_{i}^{T}P_{g}\right)\right]}^{-1} & 0\\ 0^{T} & 2{\hat{\sigma }}_{g}^{4}{\left(\sum {\hat{z}}_{g,i}\right)}^{-1} \end{array}\right],\;g=1,2,\ldots ,G. \end{array}$$

Note that the IFIM should include both the SPCR models ${\hat{F}}_{g,\mathrm{SPCR}}^{-1}$ and the time factor ${\hat{F}}_{g,\mathrm{time}}^{-1}$ for each segment.

For each segment *g*, the time factor is under the univariate Gaussian distribution. As a result, the IFIM of the time factor is

(34) $$\begin{array}{c} {\hat{F}}_{g,\mathrm{time}}^{-1}=\left[\begin{array}{cc} {\hat{\sigma }}_{g}^{2,\mathrm{time}}/n & 0\\ 0 & \frac{2}{n}{\hat{\sigma }}_{g}^{4,\mathrm{time}} \end{array}\right]. \end{array}$$

By combining the two IFIMs for the SPCR model and the time factor, we have the inverse Fisher information

(35) $$\begin{array}{c} {\hat{F}}_{g}^{-1}=\left[\begin{array}{cc} {\hat{F}}_{g,\mathrm{SPCR}}^{-1} & 0\\ 0^{T} & {\hat{F}}_{g,\mathrm{time}}^{-1} \end{array}\right]. \end{array}$$

Overall, the inverse of the estimated Fisher information matrix (IFIM) for the *MIX-SPCR* model becomes

(36) $$\begin{array}{cc} {\hat{F}}^{-1}≅\left[\begin{array}{ccccc} {\hat{F}}_{\pi }^{-1} & 0 & 0 & \cdots & 0\\ 0 & {\hat{F}}_{1}^{-1} & 0 & \cdots & 0\\ 0 & 0 & {\hat{F}}_{2}^{-1} & \cdots & 0\\ \vdots & \vdots & \vdots & \ddots & \vdots \\ 0 & 0 & 0 & \cdots & {\hat{F}}_{G}^{-1} \end{array}\right] & . \end{array}$$

Using the above definition of ICOMP(IFIM) and the properties of block-diagonal matrices with their trace and determinant, we have

(37) $$\begin{array}{c} \mathrm{ICOMP}\left(\mathrm{IFIM}\right)=-2L_{\mathrm{mix}}+2C_{1}\left({\hat{F}}^{-1}\right), \end{array}$$

where

(38) $$\begin{array}{cc} C_{1}\left({\hat{F}}^{-1}\right) & =\frac{s}{2}\log\left[\frac{tr\left({\hat{F}}_{\pi }^{-1}\right)+\sum_{g=1}^{G}tr\left({\hat{F}}_{g}^{-1}\right)}{s}\right]-\frac{1}{2}\left[\log\left|{\hat{F}}_{\pi }^{-1}\right|+\sum_{g=1}^{G}\log\left|{\hat{F}}_{g}^{-1}\right|\right], \end{array}$$

and where $s=\mathrm{rank}\left({\hat{F}}^{-1}\right)=r_{\pi }+\sum_{g=1}^{G}r_{g}=\dim\left({\hat{F}}^{-1}\right)$.

Similarly, we derive the second equivalent form of $\mathrm{ICOMP}{\left(\mathrm{IFIM}\right)}_{C_{1F}}$ as

(39) $$\begin{array}{c} \mathrm{ICOMP}{\left(\mathrm{IFIM}\right)}_{C_{1F}}=-2L_{\mathrm{mix}}+2C_{1F}\left({\hat{F}}^{-1}\right). \end{array}$$

Using the properties of the block-diagonal matrices, we have

(40) $$\begin{array}{c} tr\left({\left({\hat{F}}^{-1}\right)}^{T}\left({\hat{F}}^{-1}\right)\right)=tr{\left({\hat{F}}_{\pi }^{-1}\right)}^{2}+\sum_{g=1}^{G}tr{\left({\hat{F}}_{g}^{-1}\right)}^{2}. \end{array}$$

Thus, an open computational form of $\mathrm{ICOMP}{\left(\mathrm{IFIM}\right)}_{C_{1F}}$ becomes

(41) $$\begin{array}{c} \mathrm{ICOMP}{\left(\mathrm{IFIM}\right)}_{C_{1F}}=-2L_{\mathrm{mix}}+\frac{s}{2}\frac{\frac{1}{s}\left[tr{\left({\hat{F}}_{\pi }^{-1}\right)}^{2}+\sum_{g=1}^{G}tr{\left({\hat{F}}_{g}^{-1}\right)}^{2}\right]-{\left[\frac{tr\left({\hat{F}}_{\pi }^{-1}\right)+\sum_{g=1}^{G}tr\left({\hat{F}}_{g}^{-1}\right)}{s}\right]}^{2}}{{\left[\frac{tr\left({\hat{F}}_{\pi }^{-1}\right)+\sum_{g=1}^{G}tr\left({\hat{F}}_{g}^{-1}\right)}{s}\right]}^{2}}. \end{array}$$

We note that in computing both forms of ICOMP above, we do not need to build the full inverse of the estimated Fisher information matrix (IFIM) for the *MIX-SPCR* model given in Equation (36). All one requires is the computation of IFIM for each segment, which is appealing.

We also use AIC and CAIC (Bozdogan [33]) for comparison purposes given by

(42) $$\begin{array}{cc} \mathrm{AIC} & =-2L_{\mathrm{mix}}+2s^{*},\mathrm{and}, \end{array}$$

(43) $$\begin{array}{cc} \mathrm{CAIC} & =-2L_{\mathrm{mix}}+s^{*}\left(\log n+1\right), \end{array}$$

where $s^{*}=G\left(k+3\right)$ is the number of estimated parameters in the *MIX-SPCR* model and log denotes the natural logarithm of the sample size *n*.

Next, we show our numerical examples starting with a detailed Monte Carlo simulation study.

## 5. Monte Carlo Simulation Study

We perform numerical experiments in a unified computing environment: Ubuntu 18.04 operating system, Intel I7-8700, and 32 GB of RAM. We use the programming language Python and the scientific computing package NumPy [34] to build a computational platform. The size of the input data directly affects the running time of the program. At n=4000 time-series observations, the execution time for each EM iteration is about 0.9 s. Parameter estimation can reach convergence within 40 steps of iterations, with a total machine run time of 37 s.

### 5.1. Simulation Protocol

In this section, we present the performance of the proposed *MIX-SPCR* model using synthetic data generated from a segmented regression model. Our simulation protocol has p=12 variables and four actual latent variables. Two segmented regression models determine the dependent variable *y*, and each segment is continuous and has its own specified coefficients ($\beta_{1}$ and $\beta_{2}$). Our simulation set up is as follows:

(44) $$\begin{array}{cc} \Lambda & =\left[\begin{array}{cccc} 1.8 & 0 & 0 & 0\\ 1.8 & 0 & 0 & 0\\ 1.8 & 0 & 0 & 0\\ 0 & 1.7 & 0 & 0\\ 0 & 1.7 & 0 & 0\\ 0 & 1.7 & 0 & 0\\ 0 & 0 & 1.6 & 0\\ 0 & 0 & 1.6 & 0\\ 0 & 0 & 1.6 & 0\\ 0 & 0 & 0 & 1.5\\ 0 & 0 & 0 & 1.5\\ 0 & 0 & 0 & 1.5 \end{array}\right], \end{array}$$

(45) $$\begin{array}{cc} \psi & =\mathrm{diag}\left(1.27,0.61,0.74,0.88,0.65,0.81,0.74,1.3,1.35,0.74,0.92,1.32\right), \end{array}$$

(46) $$\begin{array}{cc} \Sigma & =\Lambda \Lambda^{T}+\psi , \end{array}$$

(47) $$\begin{array}{cc} x_{t} & ∼\mathrm{MVN}\left(0,\Sigma \right),t=1,2,\cdots ,4000, \end{array}$$

(48) $$\begin{array}{cc} \beta_{1} & =(-10,0.1,0.1,0.1,2.1,0,0,0.1,0.1,0,0,0), \end{array}$$

(49) $$\begin{array}{cc} \beta_{2} & =(0,0,0,0,0,0.5,0.3,0.1,2.1,1,2,20), \end{array}$$

(50) $$\begin{array}{cc} y_{t,g=1} & =x_{1,t}\beta_{1}+\varepsilon_{1,t},t=1,2,\cdots ,2800, \end{array}$$

(51) $$\begin{array}{cc} y_{t,g=2} & =x_{2,t}\beta_{2}+\varepsilon_{2,t},t=2801,2802,\cdots ,4000. \end{array}$$

We set the total number of time-series observations, n=4000. The first segment has $n_{1}=2800$, and the second segment has $n_{2}=1200$ time-series observations. We randomly draw error term from a Gaussian distribution with zero mean and $\sigma^{2}=9$. Among all the variables, the first six observable variables explain the first segment, and the remaining six explanatory variables primarily determine the second segment. We set the mixing proportions $\pi_{1}=0.7$ and $\pi_{2}=0.3$ for two time-series segments, respectively.

### 5.2. Detection of Structural Change Point

In the first simulation study, we limit the actual number of segments equal to two, which means that the first segment expands from the starting point to a structural change point, and the second segment expands from the change point to the end. By design, each segment is continuous on the time scale, and different sets of independent variables explain the trending and volatility. We run the *MIX-SPCR* model to see if it can successfully determine the position of the change point using the information criteria. If a change point is correctly selected, we expect that the information criteria is minimized at this change point.

**Figure 2:**
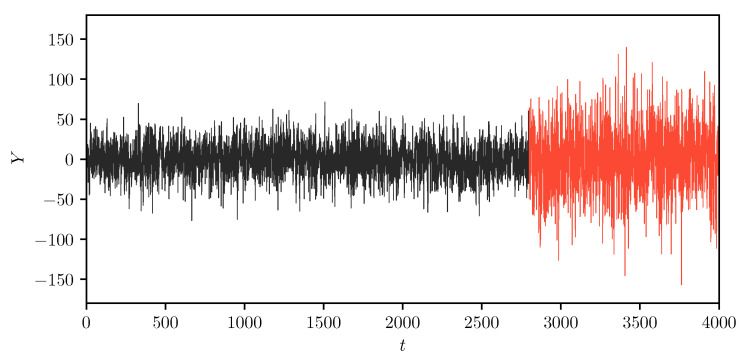
The plot of two-segment simulated time-series data. We show the plot of the simulated time-series data through the whole-time scale. Note that the first segment is from the starting point t=1 to the change point t=2800, and the second time segment expands from the change point t=2801 to the end t=4000.

**Figure 3:**
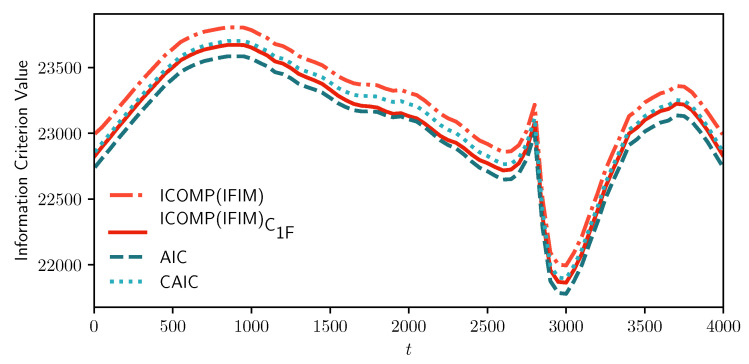
Sample path of information criteria for the simulated time-series data. The horizontal coordinate represents the position of the possible change points, and the vertical coordinate represents the corresponding information criterion (IC) values. The lower the IC value, the more likely the selected position of the change point is the real position. The real change point is t=2800.

Figure 2 and Figure 3 show our results from the *MIX-SPCR* model. Specifically, it shows the sample path of the information criteria at each time point. We note that all the information criteria values are minimized from t=2800 to t=3000, which covers the time-series’s actual change point position. As the *MIX-SPCR* model selects different change points, the penalty term of AIC and CAIC remain the same because both the number of model parameters and the number of observations do not change. In this simulation scenario, the fixed penalty term means that the AIC and CAIC reflect the changes only in the “lack of fit” term of various models without considering model complexity. This indicates that using AIC-type criteria just counting and penalizing the number of parameters may be necessary but not sufficient in model selection. As a comparison, however, we note that the penalty term of information complexity-based criteria, $C_{1}$ and $C_{1F}$, are adjusted in selecting different change points. They are varying but not fixed.

### 5.3. A Large-Scale Monte Carlo Simulation

Next, we perform a large-scale Monte Carlo simulation to illustrate the *MIX-SPCR* model’s performance in choosing the correct number of segments and the number of latent variables. A priori, in this simulation, we pretend that we do not know the actual structure of the data and use the information criteria to recover the actual construction of the *MIX-SPCR* model. To achieve this, we follow the above simulation protocol using a different number of time points by varying n=1000, 2000, 4000. As before, there are twelve explanatory variables drawn from four latent variable models generated from a multivariate Gaussian distribution given in Equation (47). The simulated data again consist of two time-series segments with mixing proportions $\pi_{1}=0.7$ and $\pi_{2}=0.3$, respectively. For each data generating process, we replicate the simulation one hundred times and record both information complexity-based criteria (ICOMP(IFIM) & $\mathrm{ICOMP}{\left(\mathrm{IFIM}\right)}_{C_{1F}}$) and classic AIC-type criteria (AIC & CAIC). In Table 1, we present how many times the *MIX-SPCR* model selects different models in the one hundred simulations. In this way, we can assess different information criteria by measuring the hit rates.

Looking at Table 1, we see that when the sample size n=1000 (small), AIC selects the correct model (G=2, k=4) 69 times, CAIC selects 80 times, ICOMP(IFIM) selects 48 times, and $\mathrm{ICOMP}{\left(\mathrm{IFIM}\right)}_{C_{1F}}$ selects 76 times, respectively, in 100 replications of the Monte Carlo simulation. When the sample size is small, ICOMP(IFIM) tends to choose a sparser regression model sensitive to the sample size. However, as the sample size increases, when n=2000 and n=4000, ICOMP(IFIM) consistently outperforms other information criteria in terms of hit rates. The percentage of the correctly identified model is above 90%, as reported above.

Our results show that the *MIX-SPCR* model works well in all settings to estimate the number of time-series segments and the number of latent variables.

**Figure 4:**
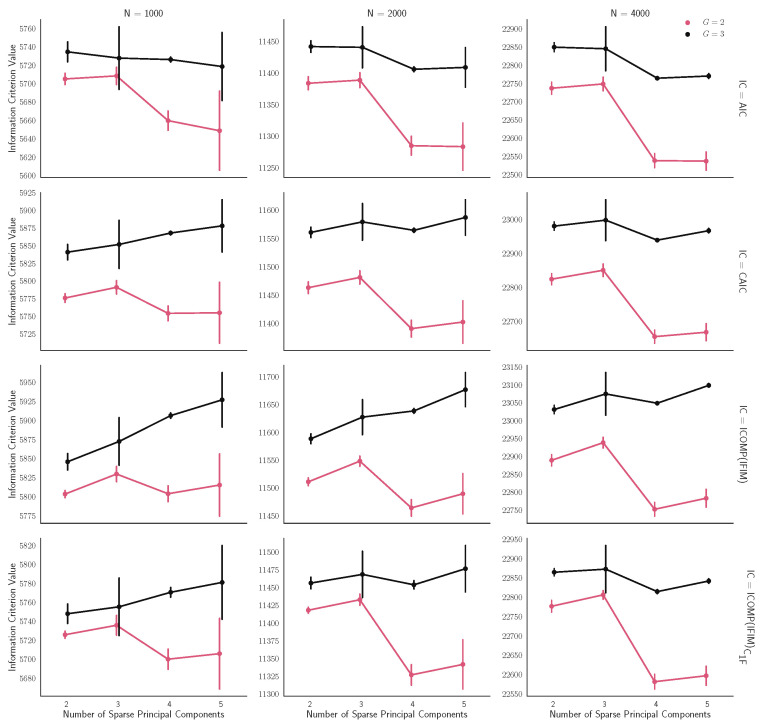
Plot of average and 1SD (standard deviation) of information criterion values over different sample sizes in all simulations with three Sparse Principal Components (SPCs) and G=2 segments. The red line indicates the estimated *MIX-SPCR* model based on two groups (G=2). Correspondingly, the black line indicates the estimated *MIX-SPCR* model for three groups (G=3). Horizontal coordinates represent different numbers of SPCs.

Figure 4 illustrates how the *MIX-SPCR* model performs if the number of segments and the number of sparse principal components are unknown beforehand.

The choice of the number of segments (*G*) has a significant impact on the results. For all the simulation scenarios, the correct choice of the number of segments (G=2) has information criterion values less than the incorrect choice (G=3). This pattern emerges consistently among all the sample sizes, both the classical ones and information-complexity based criteria.

In summary, the large-scale Monte Carlo simulation analysis highlights the performance of the *MIX-SPCR* model. As the sample size increases, the *MIX-SPCR* model improves its performance. As shown in Figure 3, the *MIX-SPCR* model can efficiently determine the structural change point and estimate the mixture proportions when the number of segments is unknown beforehand. Another key finding is that, by using the appropriate information criteria, the *MIX-SPCR* model can correctly identify the number of segments and the number of latent variables from the data. In other words, our approach can extract the main factors not only from the intercorrelated variables but also classify the data into several clearly defined segments on the time scale.

## 6. Case Study: Segmentation of the S&P 500 Index

### 6.1. Description of Data

The financial market often generates a large amount of time-series data, and in most cases, the generated data is high-dimensional. In this paper, we use the S&P 500 index and its related hundreds of company stocks categorized into eleven sectors, which are high dimensional time-series data. The index value is the response variable mixed by plenty of companies’ variations at each time point. These long time-series values often consist of different regimes and states. For example, the stock market experienced a boom period from 2017 to 2019, which is a dramatic change compared with the stock market during the 2008 financial crisis. If we analyze each sector or company, some industries perform more actively than others during a particular period.

In this section, we implement the *MIX-SPCR* model on the adjusted closing price of the S&P 500 (^GSPC) as a case study. We extract the daily adjusted closing prices from the Yahoo Finance database (https://finance.yahoo.com/) that spans the period from 1 January 1999 to 31 December 2019. By removing weekends and holidays, there are n=5292 tradable days in total. The main focus of this section is to split the time-series into several self-contained segments. Besides, we expect the extracted sparse principal components to explain the variance and volatility in each segment.

### 6.2. Computational Results

**Figure 5:**
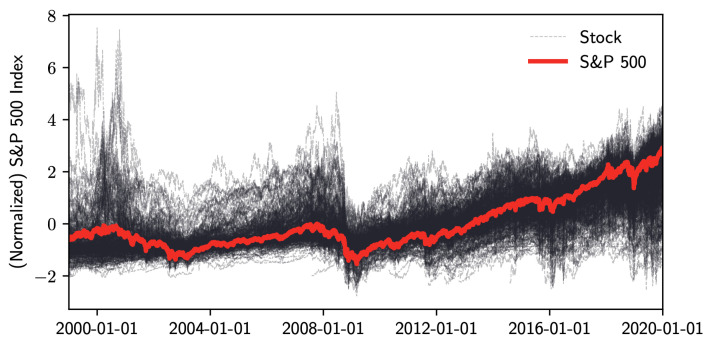
Normalized S&P 500 index and stock prices from January 1999 to December 2019.

**Figure 6:**
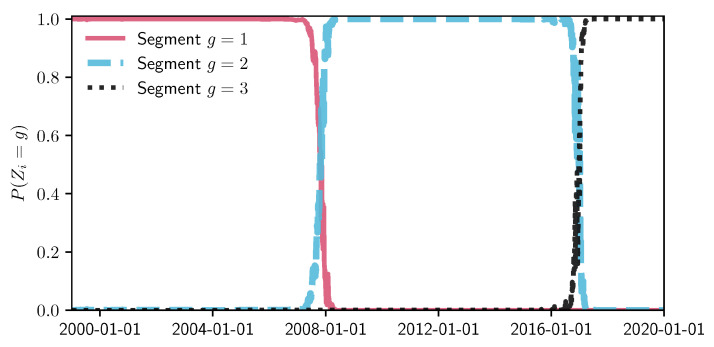
Segmented periods and probability. The plot’s vertical coordinate indicates the probability that an individual time-series data point belongs to each segment.

To have a big picture of how the S&P 500 index values reflect the changes of 506 company stock prices, Figure 5 shows the plot of the normalized values of adjusted closing prices. We use the *MIX-SPCR* model with the information criteria to determine the number of segments and the number of sparse principal components. To achieve interpretable results, we limit our search space to a maximum of seven time-series and six sparse principal components. Table 2 shows the optimal combination of three self-contained segments and three sparse principal components for each of the segments by using the information complexity ICOMP(IFIM). The other three information criteria also choose this combination as the best-fitting model. Figure 6 illustrates the probability and time range of each segment. We can see that the first segment is from 1 January 1999, to 26 October 2007. The second time-series segment spans from 29 October 2007, to the end of 2016. The last segment extends from 30 December 2016 to 31 December 2019.

We emphasize that many factors may explain the stock market variation, and this is not a research on how the socioeconomic events influence the S&P 500 index. However, it does raise our interest in the distribution of two structural change points from the segmentation results. The first change point is October 2007, which is the early stage of the 2008 financial crisis. The second structural change point is December 2016, the transitional period of the USA presidential election. Identification of these two change points shows that our proposed method can detect the underlying physical and structural change from the available time-series data.

Table 3 lists the estimated coefficients ($\beta_{g}$) from sparse principal component regression. Because all the collected stock prices and S&P 500 index values are standardized before implementing the *MIX-SPCR* model, we make dimension reduction, remove the constant term, and perform regression analysis using the SPCR model. The $R^{2}$ values are above 0.8 across all three different time segments.

### 6.3. Interpretation of Computational Results

One may ask a question, “Can the *MIX-SPCR* model identify the key variables from the hundreds of companies?” If the constructed model is dense, the selected companies would include all the sectors whereby the dense model is limiting the interpretation of the data. Our analysis identifies all the companies with non-zero coefficient values and maps them back to each of the sectors in Table A1, Table A2 and Table A3. Each calculated sparse principal component vector consists of around fifty companies, much less than the original data dimension (p=506). We observe that these selected companies are grouped into a few sectors within different time segments. For example, energy companies load in the first sparse principal component vector from 1999 to 2007 (segment 1) and diminish after that.

**Figure 7:**
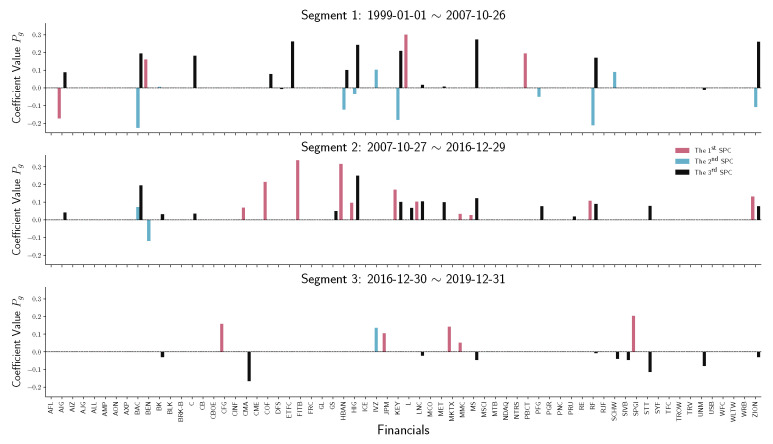
Stem plot of SPC coefficients $P_{g}$ for financial companies within each time segment. From top to bottom, the three panels represent different segmented periods, respectively. The horizontal axis of each panel indicates the company in the industrial sector. The vertical axis shows the SPC coefficient values.

**Figure 8:**
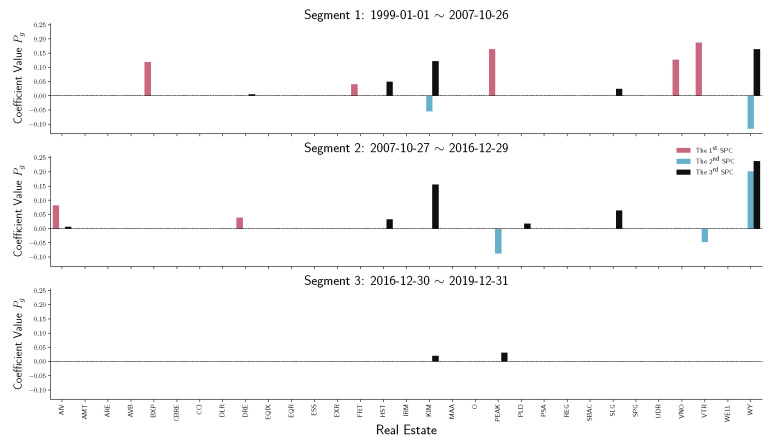
Stem plot of SPC coefficients $P_{g}$ for real estate companies within each time segment. From top to bottom, the three panels represent different segmented periods, respectively. The horizontal axis of each panel indicates the company in the industrial sector. The vertical axis shows the SPC coefficient values.

**Figure 9:**
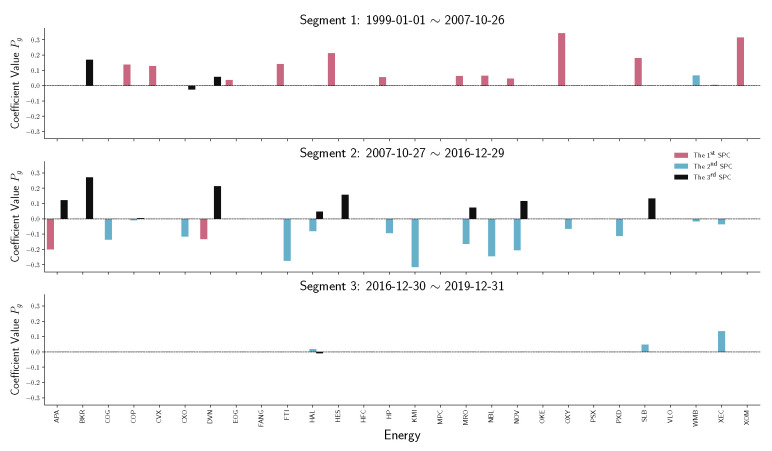
Stem plot of SPC coefficients $P_{g}$ for energy companies within each time segment. From top to bottom, the three panels represent different segmented periods, respectively. The horizontal axis of each panel indicates the company in the industrial sector. The vertical axis shows the SPC coefficient values.

To have a detailed analysis of how different sectors perform across three segments, we do the stem plot to show the sparse principal component coefficients $P_{g}$ of four sectors, namely financials, real estate, energy, and information technology (IT). Figure 7 and Figure 8 indicate a similar behavior that happened in financial and real estate companies. Both sectors play an essential role in the first two time-series segments but have no contribution in the third segment, which is the period after December 2016. Notice that in Figure 9, energy companies act as an essential player before 2016. However, during the recession in 2008, energy company loadings are negated from the first SPC to the second SPC. Compared with other industries, the variation in energy company stock prices does not contribute to the S&P 500 index after 2016.

**Figure 10:**
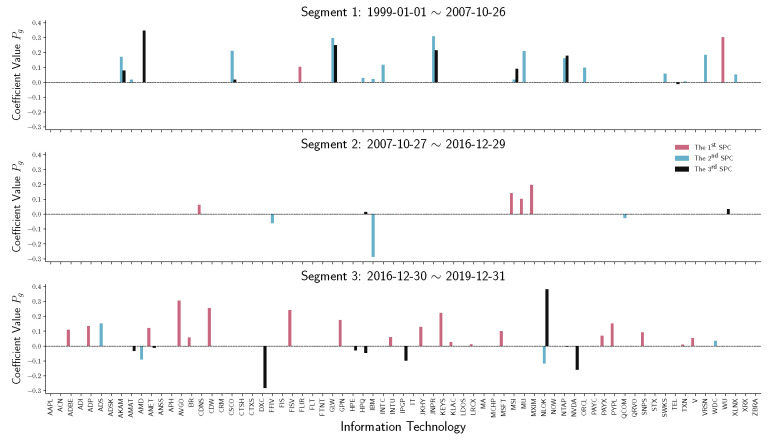
Stem plot of SPC coefficients $P_{g}$ for information technology companies within each time segment. From top to bottom, the three panels represent different segmented periods, respectively. The horizontal axis of each panel indicates the company in the industrial sector. The vertical axis shows the SPC coefficient values.

Another question is ”What sector/industry is the main contributing factor after the 2016 United States presidential election?” A possible answer is, as shown in Figure 10, the SPC coefficients of information technology companies. From 1999 to the recession in 2008, IT companies work mainly on the second SPC and the third SPC, which do not contribute much to the main variation. After the recession, the variations of IT companies do not contribute compared with other sectors. However, after December 2016, companies from the IT industry play an essential role in the primary stock price volatility.

**Figure 11:**
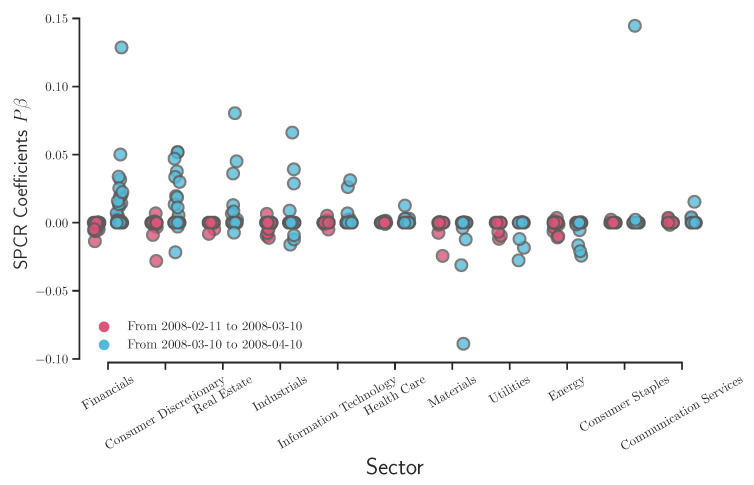
Overlay plot of the SPCR coefficients before/after 2008 financial crisis.

As discussed above, Figure 7, Figure 8, Figure 9 and Figure 10 provide a clear picture of how different sectors perform (via coefficient $P_{g}$) without considering the effects on the S&P 500 index. It might raise the interest in how the SPCR coefficient $P_{g}\beta_{g}$ changes before/after certain socioeconomic events. We follow the research implemented by Aït-Sahalia and Xiu [35] about how the Federal Reserve addressing heightened liquidity from March 10 to 14 March 2008, affects the stock market. The data analyzed by Aït-Sahalia and Xiu [35] are the S&P 100 index values using the traditional PCA, and the authors grouped stocks into financial and non-financial categories. Instead of PCA, we apply the SPCR model on the S&P 500 index and analyze how eleven sectors react before/after Federal Reserve operations. Figure 11 shows that financials, consumer discretionary, real estate, and industrials experienced more significant perturbations than other sectors in terms of SPCR coefficients $P_{g}\beta_{g}$. This conclusion is consistent with the results from Aït-Sahalia and Xiu [35] that the average loadings of first and second principal components of financial companies are distinct from non-financial companies. However, considering that we have 506 companies in the raw data and make a sparse loading of companies for comparison, the excessive explanatory power is still maintained in this high-dimensional case using the SPCR model, which is more interpretable.

## 7. Conclusions and Discussions

In this paper, we presented a new and novel method to segment high-dimensional time-series data into different clusters or segments using the mixture model of the sparse principal components model (*MIX-SPCR*). The *MIX-SPCR* model considers both the relationships among the predictor variables and how various predictor variables contribute the explanatory power to the response variable through the sparsity settings. Information criteria have been introduced and derived for the *MIX-SPCR* model. These criteria are applied to study their performance under different sample sizes and to select the best-fitting model.

Our large-scale Monte Carlo simulation exercise showed that the *MIX-SPCR* model could successfully identify the real structure of the time-series data using the information criteria as the fitness function. In particular, based on our results, the information complexity-based criteria—i.e., ICOMP(IFIM) and $\mathrm{ICOMP}{\left(\mathrm{IFIM}\right)}_{C_{1F}}$—outperformed the conventional standard information criteria, such as the AIC-type criteria as the data dimension and the sample size increase.

Later, we empirically applied the *MIX-SPCR* model to uncover the S&P 500 index data (from 1999 to 2019) and identify two change points of this data set.

We observe that the first change point physically coincides with the early stages of the 2008 financial crisis. The second change point is immediately after the 2016 United States presidential election. This structural change point coincides with the election of President Trump and his transition.

Our findings showed how the S&P 500 index and company stock prices react within each time-series segment. The *MIX-SPCR* model presents excessive explanatory power by identifying how different sectors fluctuated before/after the Federal Reserve’s addressing heightened liquidity from 10 March to 14 March 2008.

Although this is not a traditional event study paper, it is the first paper to use the sparse principal component regression model with mixture models in the time-series analysis. The proposed new and novel *MIX-SPCR* model enlightens us to explore more interpretable results on how macroeconomic factors/events influence the stock prices on the time scale. Later, in a separate paper, we will incorporate the event study in the *MIX-SPCR* model as our future research initiative.

This paper’s time segmentation model builds on time-series data, constructs likelihood functions, and performs parameter estimation by introducing error information unique to each period. Researchers have recently realized that environmental background noise can positively affect the model building and analysis under certain circumstances ([36,37,38,39,40,41,42]). For example, in Azpeitia and Wagner [40], the authors highlighted that the introduction of noise is necessary to obtain information about the system. In our next study, we would like to explore this positive effect of environmental noise even further and use it to build better statistical models for analyzing high-dimensional time-series data.

## Figures and Tables

**Table 1:** Frequency of the choice of the true model with information criteria in 100 replications of the experiment for each sample size (*n*) of time-series observations. The true model is G=2 and k=4.

		n=1000		n=2000		n=4000	
		G = 2	G = 3	G = 2	G = 3	G = 2	G = 3
AIC	k = 2	0	0	0	0	0	0
	k = 3	0	6	0	3	0	1
	k = 4	69	0	77	0	75	0
	k = 5	24	1	20	0	24	0
CAIC	k = 2	1	0	0	0	0	0
	k = 3	1	3	0	1	0	1
	k = 4	80	0	96	0	93	0
	k = 5	14	1	3	0	6	0
ICOMP(IFIM)	k = 2	31	2	1	0	0	0
	k = 3	2	5	0	2	0	1
	k = 4	48	0	96	0	96	0
	k = 5	11	1	1	0	3	0
$\mathrm{ICOMP}{\left(\mathrm{IFIM}\right)}_{C_{1F}}$	k = 2	2	1	0	0	0	0
	k = 3	0	7	0	3	0	1
	k = 4	76	0	93	0	93	0
	k = 5	13	1	4	0	6	0

**Table 2:** The ICOMP(IFIM) values of segmentation results for S&P 500 index data (Lower is better).

		Number of Sparse Principal Components				
		1	2	3	4	5
Number of Segments	1	30,097.04	30,092.45	30,106.50	30,121.64	30,145.13
	2	29,975.01	30,058.40	30,293.55	30,234.65	30,347.94
	3	30,010.70	30,062.19	29,241.52	30,453.74	30,526.20
	4	29,877.27	29,825.73	29,811.53	30,571.39	30,628.61
	5	29,904.35	29,973.47	30,011.18	30,311.52	30,554.82
	6	30,111.35	30,361.39	30,388.47	30,665.26	30,581.29
	7	30,031.39	30,564.65	30,597.14	30,823.76	31,057.54

**Table 3:** SPCR coefficients ($\beta_{g}$) of three different segments.

	Segment 1 ($R^{2}=0.82$)	Segment 2 ($R^{2}=0.94$)	Segment 3 ($R^{2}=0.97$)
	01-01-1999 ∼ 26-10-2007	27-10-2007 ∼ 29-12-2016	30-12-2016 ∼ 31-12-2019
SPC1	0.0964	0.1240	0.1512
SPC2	0.0729	−0.0439	0.0359
SPC3	0.0079	0.0191	−0.0051

## Abbreviations

The following abbreviations are used in this manuscript:

*MIX-SPCR*	Mixture of the sparse principal component regression model
CV	Cross validation
TICC	Toeplitz inverse covariance-based clustering
GGS	Greedy Gaussian Segmentation
PCA	Principal Component Analysis
PC	Principal Component
SPCA	Sparse Principal Component Analysis
SPCR	Sparse Principal Component Regression
SPC	Sparse Principal Component
SPPCA	Sparse Probabilistic Principal Component Analysis
EM	Expectation–Maximization (Algorithm)
IC	Information Criterion
ICOMP	Information Complexity

## Appendix A. Tables

**Table A1:** Sparse Principal Component (SPC) of Segment 1 (1 January 1999 ∼ 26 October 2007).

	SPC1		SPC2		SPC3	
	Count	Percentage	Count	Percentage	Count	Percentage
Health Care	4	6.56	6	9.84	1	1.64
Industrials	6	8.57	3	4.29	6	8.57
Utilities	5	17.86	3	10.71	3	10.71
Materials	2	7.14	1	3.57	1	3.57
Consumer Discretionary	5	7.81	6	9.38	6	9.38
Energy	13	46.43	1	3.57	3	10.71
Financials	5	7.58	10	15.15	15	22.73
Real Estate	5	16.13	2	6.45	5	16.13
Consumer Staples	2	6.06	0	0.00	1	3.03
Communication Services	1	3.85	2	7.69	1	3.85
Information Technology	2	2.82	16	22.54	8	11.27

**Table A2:** Sparse Principal Component (SPC) of Segment 2 (27 October 2007 ∼ 29 Decmeber 2016).

	SPC1		SPC2		SPC3	
	Count	Percentage	Count	Percentage	Count	Percentage
Health Care	7	11.48	2	3.28	1	1.64
Industrials	5	7.14	6	8.57	4	5.71
Utilities	0	0.00	0	0.00	5	17.86
Materials	6	21.43	2	7.14	3	10.71
Consumer Discretionary	7	10.94	14	21.88	3	4.69
Energy	2	7.14	14	50.00	9	32.14
Financials	12	18.18	3	4.55	16	24.24
Real Estate	2	6.45	3	9.68	6	19.35
Consumer Staples	0	0.00	0	0.00	0	0.00
Communication Services	5	19.23	3	11.54	1	3.85
Information Technology	4	5.63	3	4.23	2	2.82

**Table A3:** Sparse Principal Component (SPC) of Segment 3 (30 Decmeber 2016 ∼ 31 Decmeber 2019).

	SPC1		SPC2		SPC3	
	Count	Percentage	Count	Percentage	Count	Percentage
Health Care	10	16.39	14	22.95	2	3.28
Industrials	9	12.86	4	5.71	4	5.71
Utilities	1	3.57	0	0.00	0	0.00
Materials	1	3.57	3	10.71	6	21.43
Consumer Discretionary	3	4.69	10	15.63	8	12.50
Energy	0	0.00	3	10.71	1	3.57
Financials	5	7.58	1	1.52	10	15.15
Real Estate	0	0.00	0	0.00	2	6.45
Consumer Staples	0	0.00	6	18.18	3	9.09
Communication Services	2	7.69	5	19.23	5	19.23
Information Technology	19	26.76	4	5.63	9	12.68
